# Evaluating resective surgery targets in epilepsy patients: A comparison of quantitative EEG methods

**DOI:** 10.1016/j.jneumeth.2018.04.021

**Published:** 2018-07-15

**Authors:** Michael Müller, Kaspar Schindler, Marc Goodfellow, Claudio Pollo, Christian Rummel, Andreas Steimer

**Affiliations:** a Department of Neurology, Inselspital, Bern University Hospital, University Bern, Bern, Switzerland; b Support Center for Advanced Neuroimaging (SCAN), University Institute for Diagnostic and Interventional Neuroradiology, Inselspital, Bern, Switzerland; c College of Engineering, Mathematics and Physical Sciences, University of Exeter, Exeter, UK; d Centre for Biomedical Modelling and Analysis, University of Exeter, Exeter, UK; e EPSRC Centre for Predictive Modelling in Healthcare, University of Exeter, Exeter, UK; f Department of Neurosurgery, Inselspital, Bern University Hospital, University Bern, Bern, Switzerland

**Keywords:** Epilepsy, Quantitative EEG, Resective surgery, Predictive modeling, Functional network, Method validation

## Abstract

**Background:**

Quantitative analysis of intracranial EEG is a promising tool to assist clinicians in the planning of resective brain surgery in patients suffering from pharmacoresistant epilepsies. Quantifying the accuracy of such tools, however, is nontrivial as a ground truth to verify predictions about hypothetical resections is missing.

**New method:**

As one possibility to address this, we use customized hypotheses tests to examine the agreement of the methods on a common set of patients. One method uses machine learning techniques to enable the predictive modeling of EEG time series. The other estimates nonlinear interrelation between EEG channels. Both methods were independently shown to distinguish patients with excellent post-surgical outcome (Engel class I) from those without improvement (Engel class IV) when assessing the electrodes associated with the tissue that was actually resected during brain surgery. Using the AND and OR conjunction of both methods we evaluate the performance gain that can be expected when combining them.

**Results:**

Both methods’ assessments correlate strongly positively with the similarity between a hypothetical resection and the corresponding actual resection in class I patients. Moreover, the Spearman rank correlation between the methods’ patient rankings is significantly positive.

**Comparison with existing method(s):**

To our best knowledge, this is the first study comparing surgery target assessments from fundamentally differing techniques.

**Conclusions:**

Although conceptually completely independent, there is a relation between the predictions obtained from both methods. Their broad consensus supports their application in clinical practice to provide physicians additional information in the process of presurgical evaluation.

## Highlights

• Quantitative EEG methods have potential to provide clinically relevant information.
• Two examined methods correctly depict the effects of resective epilepsy surgery.
• Their broad consensus supports their application in presurgical evaluation.
• Cross-method validation could help overcome the task's missing ground truth.

## 1 Introduction

Epilepsy is one of the most prevalent neurological disorders and affects at least 50 million people worldwide (World Health Organization, 2001). In approximately one third of all patients seizure freedom is not achieved by pharmaceutical therapies and in these cases surgical treatment should then be considered. The goal of epilepsy surgery is to selectively resect brain tissue with the aim that this procedure renders the patient seizure free. However, there is currently no diagnostic method to unequivocally delineate the neuroanatomical areas that are necessary and sufficient to generate epileptic seizures, the epileptogenic zone (EZ) (Rosenow and Lüders, 2001, Lüders et al., 2006). Instead, the area showing first ictal epileptiform EEG signals (the seizure onset zone, SOZ) is often used in clinical practice as a proxy for the EZ, since the SOZ is thought to overlap with the EZ (Rosenow and Lüders, 2001). However, the exact boundaries of the SOZ and the actual extent of overlap with the EZ for any given patient remain unknown. Moreover, a recent study found that to attain seizure freedom, complete resection of the SOZ was necessary in only one out of eight pediatric patients (Huang et al., 2012). Together with evidence that long-term seizure freedom is only achieved in up to 2/3 of patients who undergo surgery (Wiebe et al., 2001, Téllez-Zenteno et al., 2005, de Tisi et al., 2011, Engel et al., 2012), doubt can be cast regarding whether the SOZ is the best approximation to the EZ, or whether alternative methods to identify which regions of tissue to resect could be beneficial. An additional challenge to the use of the SOZ is that it is determined predominantly by visual analysis of EEG recordings, which is not only time consuming but also prone to inter-rater variability.

To address these shortcomings, a variety of quantitative intracranial EEG (iEEG) analysis methods have been developed to aid identification of candidate tissue for surgical resection. Many different approaches are used to assign estimates about epileptogenicity of brain tissues associated with specific channels of intracranial electrodes (see e.g. Pereda et al., 2005, Lehnertz et al., 2009, Wendling et al., 2010, van Mierlo et al., 2014). Some studies examined the relation of quantitatively determined channels with the channels determined visually as the site of seizure onset (see e.g. Urrestarazu et al., 2007, Worrell et al., 2008, Jacobs et al., 2009, Gnatkovsky et al., 2011, Gnatkovsky et al., 2014, Boido et al., 2014, Geier et al., 2015). Others explicitly verified the potential of quantitative measures to act as biomarkers of the epileptogenic zone by its relation with the actually resected brain tissue or the post-surgical seizure control. Some by capturing high-frequency oscillations (see e.g. Jacobs et al., 2010, Wu et al., 2010, Modur et al., 2011, Park et al., 2012, Roehri et al., 2017), others using graph theory to determine nodes’ values of connectivity, centrality or similar (see e.g. Jung et al., 2011, Zubler et al., 2015, Wilke et al., 2011, van Mierlo et al., 2013) and also different techniques (see e.g. Bartolomei et al., 2008, Kim et al., 2014, Kim et al., 2010). Many of these methods have shown to provide useful information in the preoperative process. Rummel et al. recently investigated how post-operative seizure control is associated with different qEEG measures representative for four different classes of signal analysis methods (Rummel et al., 2015). They calculated four different measures and salient channels were selected by a data-driven manner for each measure. For three of these measures, the overlap between salient channels and actually resected channels was significantly larger for class I patients compared to class IV patients. A measure derived from a nonlinear interrelation matrix could best differentiate between actual resections with favorable and unfavorable outcome by identifying their overlap with the channels associated with the resected brain tissue.

Computational models capable of drawing inferences about specific hypothetical resections under modifiable input conditions have been developed rather recently. Hutchings et al. used diffusion tensor imaging data and showed their model to successfully identify regions known to be involved in temporal lobe epilepsy (TLE), however, it was not validated with actual patient outcomes (Hutchings et al., 2015). Sinha et al. used interictal electrographic recordings to generate their model, which then in simulated resections showed agreement with the clinical outcome for five of six patients (Sinha et al., 2014). These two models allow to make predictions on the ictogenicity of individual nodes of a derived network. Sinha et al. recently extended their approach to make predictions about the overall efficacy of a surgical resection by averaging the seizure likelihood of all nodes under a resection and comparing it to the average obtained from random resections (Sinha et al., 2016). When simulating the actual resections the predicted outcomes coincided with the actual outcomes in 13 of 16 patients. Goodfellow et al. introduced a model that is able to quantify local and global ictogenicity of a network under perturbations of specific nodes (Goodfellow et al., 2016). They found that the overlap between resected tissue and the nodes having the biggest ictogenicities is significantly larger in patients with good response to surgery than in class IV patients. Furthermore, the model predicts a greater reduction in network ictogenicity when simulating actual resections of class I patients than for class IV patients. Based on the global network ictogenicity they classified correctly 14 out of 16 patients (AUC = 0.87). Steimer et al. presented a distributional, soft clustering model for the predictive modeling of multivariate, peri-ictal iEEG time series (Steimer et al., 2017). This model permits patient-specific predictions about seizure propensity under arbitrary simulated resections of brain tissue. Whereas the simulated resection of the brain areas that were actually surgically removed reduces the model's seizure probability in most Engel class I patients, for most Engel class IV patients the model confirms the inefficiency of the actual resection to impede an imminent seizure. Moreover, successful actual resections are significantly separated from unsuccessful ones and from equally-sized random resections while unsuccessful actual resections cannot be separated from random resections.

The availability of many alternative methods to predict which tissue should be resected raises the issue of selecting an appropriate method for a given patient. Unfortunately, because the true effect of all possible resections except those actually carried out cannot be known, determining accuracies of such methods is always restricted to very few data points and thus remains vague. A starting point to address this is to explicitly compare predictions arising from different methods and quantify, in the first instance, to what extent predictions differ, if at all. Providing a framework to answer this question would significantly advance the clinical usefulness of quantitative methods in epilepsy surgery and other treatments for neurological and neuropsychiatric disorders more generally.

For this cross-method verification of two fundamentally differing methods we focus on comparing two methods that have recently been developed and tested at our institute and have shown convincing performances by quantitative comparison with the actual resection and outcome in patients undergoing surgery. That is, we directly compare the assessments of hypothetical resections by the nonlinear interrelation measure examined by Rummel et al. (2015) with the resections’ seizure suppressing efficiencies as estimated by the model of Steimer et al. (2017). Both methods have shown promise in the prediction of tissue resection in epilepsy surgery. However, it remains unclear if their predictions are coherent beyond the common feature that successful actual resections are recognized as effective and thus get high performances. To investigate the extent to which predictions from these methods are in agreement, we compare in a first part the individual performances of the two methods for a common set of patients. In addition, we examined the performance gain that can be expected when combining the methods’ binary classifiers. In a second part we present the results of the investigation looking for a link between these methods’ classification of arbitrary resections. Finally, we discuss the obtained results and address issues of possible future work aiming to derive objective markers of target tissue or to assess such approaches.

## 2 Methods

### 2.1 Patients & data

In this study we included the peri-ictal intracranial EEG recordings of 20 patients of the epilepsy surgery program of the Inselspital Bern (15 female, 5 male; median age 31 years, IQR 16 year, range 10–66 years). A precondition for the selection of patients was the availability of the information about the resected brain tissue (incl. the associated electrodes) and their outcome according to the Engel classification scheme (Engel et al., 1993). We included patients who were post-surgically free of disabling seizures and auras for at least one year (Engel class I) or who showed no worthwhile improvement following resection (Engel class IV). All patients are listed with further details in Table 1.

**Table 1:** Patients included in this study.

Patient	Engel class	Syndrome	Etiology/MRI/Histology	# of el.	# of res. el.	# of epi. el.	Patient label in	
							Steimer et al. (2017)	Rummel et al. (2015)
1	I	MTLE (R)	Non-lesional	64	20	49	9	I-1
2	I	MTLE (L)	Hippocampal sclerosis	64	13	51	6	I-2
3	I	LTLE (L)	Cluster of dysplastic neurons	56	5	56	–	I-3
4	I	PLE (L)	Low-grade glioma	74	13	2	4	I-4
5	I	MTLE (L)	Hippocampal sclerosis	42	11	40	2	I-5
6	I	FLE (R)	Non-lesional	98	11	98	1	I-6
7	I	TLE (L)	Non-lesional	60	11	60	7	–
8	I	PLE (R)	Non-lesional	68	13	67	10	–
9	I	MTLE (R)	Hippocampal sclerosis	37	9	2	–	–
10	I	MTLE (L)	Hippocampal atrophy	31	7	31	–	–
11	I	MTLE (R)	Hippocampal sclerosis	38	8	4	–	–
12	I	FLE (L)	Non-lesional	76	7	75	–	–
13	I	FTE (R)	Aneurysmal subarachnoid haemorrhage	80	6	17	–	–
14	IV	LTLE (L)	Dysplasia	59	2	20	5	IV-1
15	IV	LTLE (L)	Meningitis	61	10	61	8	IV-2
16	IV	MTLE (L)	Suspected amygdala dysplasia	49	8	13	18	IV-3
17	IV	PLE (L)	Non-lesional	62	4	0	21	IV-4
18	IV	FLE (R)	Tuberous sclerosis	36	3	23	NP	IV-5
19	IV	LTLE (L)	Temporo-basal dysplasia	24	6	22	–	–
20	IV	FLE (L)	Non-lesional	69	4	68	–	–

Indicated is the outcome of the resective surgery according to the Engel classification scheme, the syndrome, laterality and etiology, the number of implanted electrodes (el.), the number of electrodes associated with resected brain tissue (res. el.) and the number of electrodes showing epileptiform activity at least 10% of the total seizure time (epi. el.). For easier comparison with earlier publications the labels used in Steimer et al. (2017) and Rummel et al. (2015) are also given (hyphen means this patient was not used in the respective publication). Abbreviations: MTLE: mesial temporal lobe epilepsy, LTLE: lateral temporal lobe epilepsy, PLE: parietal lobe epilepsy, FLE: frontal lobe epilepsy, TLE: temporal lobe epilepsy, FTE: fronto-temporal epilepsy, R: right, L: left.

All recordings were visually inspected by an experienced epileptologist/electroencephalographer (K.S.) to remove channels exhibiting permanent artifacts (<5% of channels) and to determine the clinical seizure onset (the time of earliest EEG change associated with seizures) and its corresponding zone (SOZ). Furthermore, pre- and post-operative MR images and post-implantation CT images were coregistered to identify the resected brain tissue and the position of the electrodes and thereby the channels recording from the subsequently resected tissue. These channels constitute the actual resection. A more detailed description of this procedure can be found in Rummel et al. (2015). In addition, the number of channels showing epileptiform activity at least 10% of the total seizure time was determined according to the channel-wise absolute signal slope as described in detail in Schindler et al. (2007). Due to the fast low-amplitude and slow high-amplitude EEG activity at the onset of and during intracranially recorded seizures this quantity increases and is thus an appropriate marker of epileptiform activity.

As argued in detail in Steimer et al. (2017), since patients are supplied again with seizure suppressing medication after resection, early recordings are presumably more representative for the postoperative state because remnants of the medication (withdrawn after implantation) may still be potent. Therefore we used the first occurring seizure after implantation except for patients 8 and 13 where we used the second seizure because the first one was corrupted by artifacts. In both examined approaches the intracranial EEG data is used at a sampling rate of 512 Hz, re-referenced against the median of all artifact-free channels, band-pass filtered between 0.5 and 150 Hz using a fourth-order Butterworth filter (applying forward and backward filtering to minimize phase shift) and then subdivided into consecutive overlapping windows. Further preprocessing steps of both approaches are specified in their descriptions in Appendix A.

Retrospective data analysis had been approved by the ethics committee of the Canton of Bern/Switzerland. All patients gave written informed consent that their EEG data may be used for research purposes.

### 2.2 Distributional soft clustering of multivariate time series

The goal of this approach is to characterize certain signal dynamics that are representative for different epochs of the peri-ictal segment of an EEG recording. These particular dynamics, stored as states of a model generated based on the EEG recording, ideally represent different brain states (e.g. interictal, seizure onset, etc.). The states that are active during the seizure are considered the ictal states while the others are the non-ictal states. The model also specifies the probabilities of all states to emerge from any other state. The models were generated on a peri-ictal part of the iEEG recordings including the complete seizure and the preceding 180s of the preictal period. It is necessary to include preictal data to allow the model to learn non-ictal states and the transition to ictal states (seizures). A more detailed description of this method can be found in Appendix A.1.

With this data-specific model, it is possible to predict how probable each representative state is for a given time point under changeable input conditions. Phrased differently, it is possible to alter the input signals and the model predicts how the system's dynamics evolve from a given time point on. In the present case, altering the input signals means simulating resections of certain brain regions (by eliminating the input signals of the electrodes associated with these regions), and predicting the future dynamics means giving the probability of developing ictal states. We used the same performance measure to rate simulated resections as introduced by Steimer et al. (2017). Accordingly, when talking about this soft clustering (SC) approach, performance of a resection describes how much more probable the model remains in a non-ictal state under this very resection than without any resection. In Eq. (1) (cf. Eq. 2.2 in Steimer et al., 2017), 〈*p*_*no*,*ict*_〉 is the summed probability of all ictal states when no channels are virtually resected, 〈*p*_*res*,*ict*_〉 is the summed probability of all ictal states when a resection *res* is simulated and 〈*p*_*res*,*ict*_〉_*norm*_ is the normalized dynamical outcome performance that is used as performance measure of the soft clustering approach (subsequently referred to as *SC performance*):

(1) $$\langle p_{\mathrm{res},\mathrm{ict}}\rangle_{\mathrm{norm}}≔\frac{\langle p_{\mathrm{no},\mathrm{ict}}\rangle -\langle p_{\mathrm{res},\mathrm{ict}}\rangle }{\langle p_{\mathrm{no},\mathrm{ict}}\rangle }$$

### 2.3 Multivariate nonlinear interrelation based functional networks

This approach defines functional networks with a patient's EEG channels as nodes and the edges defined by their nonlinear interrelations. As a measure of nonlinear interrelation, we used mutual information. In order to generate assessments of resections, it is necessary to quantify some property of each node of the functional network. As suggested in Rummel et al. (2015) we used the node strength of the functional connectivity matrix as channel-wise quantifier of nonlinear interrelation. For this approach, we considered data from the first half of a seizure since this segment of the underlying data has been shown to contain information relevant for the prediction of surgical outcomes (Rummel et al., 2015, Goodfellow et al., 2016). A more detailed description of the derivation of the mutual information matrix and the node strength can be found in Appendix A.2.

When talking about the functional network (FN) approach, the performance of a resection is the fraction of nonlinear interrelation (specified by the node strength) that is present in the channels of this resection. In Eq. (2), *n* is the number of channels and channel *i* has node strength *s*_*i*_. For a virtual resection *res*, the collective node strength *s*_*res*_ is the sum of the channels’ individual values in that resection divided by the summed node strength of all channels. So the performance measure of this approach is proportional to the fraction of the total node strength that is comprised by a virtual resection (subsequently referred to as *FN performance*):

(2) $$s_{\mathrm{res}}≔\frac{\sum_{i\in \mathrm{res}}s_{i}}{\sum_{i=1}^{n}s_{i}}$$

Since the distribution of node strength across channels and the number of channels in a resection vary between patients, each patient's distribution of performances of non-overlapping random resections was normalized to have a mean of 1 and the values of the actual resections and the overlapping resections were adjusted with the respective patient's normalizing factor (see Section 2.4 for details on random resections). This simplifies comparison and aggregation of results, however, *FN preformance* values can consequently not be interpreted as the standardized fraction of the total node strength.

### 2.4 Comparison of methods

The main goal of this study was to investigate, to what extent two different quantitative analysis methods rate resections similarly. To address this question we sought to study not only the actual resections that were performed, but also a suite of hypothetical resections. This allows us to assess more generally whether insight would differ across different methods. As our sample sizes (patient numbers) are small and underlying distributions of measures are unknown, we used bootstrapping to determine the significance of test results. For each test we generated appropriate data that constituted the distribution of the test statistic under the null hypothesis and the relative position of the actual data in this distribution determined the corresponding *p*-value. Specification of each test's null hypothesis and a detailed description of what is done in every performed test using bootstrapping can be found in Appendix B. This procedure is distribution-independent and takes the possibility of sporadic samples not representative of the population into consideration (Adèr et al., 2008). The significance level *α* for all tests was 0.05.

To compare methods applied to patients’ actual resections we examined how each measure separates the two outcome groups. We tested whether the mean ratings of the actual resections of both groups are equal (see Appendix B.1 for test details). We further assessed the extent to which the two measures yielded equivalent rankings for patients, in terms of the magnitude of “performance”. We did this by computing Spearman's rank correlation coefficient between the resulting patient ranks derived from both methods (see also Appendix B.2). We also calculated each method's performance as binary classifier by computing the receiver operating characteristic (ROC) and the corresponding area under the curve (AUC). In addition, we examined how decisions about the benefit of resections were influenced when the separate binary classifier performances of both methods were combined. To do so, we determined the optimal binary classifiers by setting the threshold according to the point on the ROC-curve with minimal distance to 100% sensitivity and specificity and combined them by an AND-conjunction (resections are only assessed as beneficial if both methods agree on it) and by an OR-conjunction (resections are assessed as beneficial if at least one method concludes so). Then, we counted for all classifiers the correct and incorrect classifications and calculated the corresponding sensitivities, specificities, and positive and negative predictive values. In this context true positives and true negatives are correctly classified beneficial resp. not beneficial resections, false negatives are resections assessed as not beneficial although they rendered the patient seizure free in reality and false positives are resections assessed as beneficial although they did not have any curative effect in reality. A similar procedure was applied to seizure prediction algorithms and found to increase the classification performance (Feldwisch-Drentrup et al., 2010).

In order to extend our insight into the performance and comparison of our methods beyond resections that were actually performed, we generated a suite of artificial resections. For each patient we created two different sets of random resections of equivalent size to the actual resection: 3000 random resections were not allowed to overlap with the actual resection and 300 random resections were specified to overlap with the actual resection in a varying number of channels. Using the non-overlapping random resections of all patients, we determined how likely the distribution of performances of these resections overlapped with the actual resections of each outcome class. In order to do this we used the L1-based distance between the cumulative distribution functions to measure their similarity (see also Appendix B.3).

Using overlapping random resections, we determined to what extent the rating of a random resection depends on its overlap (in terms of channels) with the actual resection. Let the number of channels in the actual resection be *m*. We generated *m* groups, each containing ⌊300/*m*⌋ random resections, and the resections of every group overlap with the actual resection in a number of channels between 0 and *m* − 1. All random resections were then evaluated by both methods and we determined the dependence of resection ratings on their overlap with the actual resection. We quantified this dependence with Pearson's product-moment correlation coefficient and checked for a significant difference between class I and class IV patients (see also Appendix B.4). To determine the dependences between ratings and overlaps for groups of patients, an additional step is necessary because the actual resections of different patients contain different numbers of channels. First, we transformed the size of every resections’ overlap to its fraction of the corresponding actual resection. According to their overlap fractions, we then split all virtual resections of the selected patients into 9 bins between 0 and 1 (nine is the mean size of all actual resections). Consequently, if a patient's actual resection contains less than nine channels, its virtual resections do not contribute to all bins and vice versa, if the actual resection contains more than nine channels, some virtual resections with different overlaps contribute to the same bin. In this way, it is possible to observe the same characteristic as before but for groups of patients, namely class I and class IV patients. We then determined separately for both outcome classes the Pearson's product-moment correlation coefficients between the bin-wise mean ratings of both methods (including the actual resections as an additional bin representing full overlap) and the overlap (the bin centers). In addition we calculated the correlation coefficient and its significance among the ratings (see also Appendix B.5). To determine the relation between the methods’ ratings excluding the overlap as an explanatory variable, we used the concept of partial correlation. We computed the residuals of both ratings using the overlap as regressor and then determined the correlation coefficient between these residuals. This allows us to assess the conditional independence of the ratings, that is, if there is a direct dependence among them or only via the overlap as a third variable (see also Appendix B.5).

## 3 Results

**Fig. 1:**
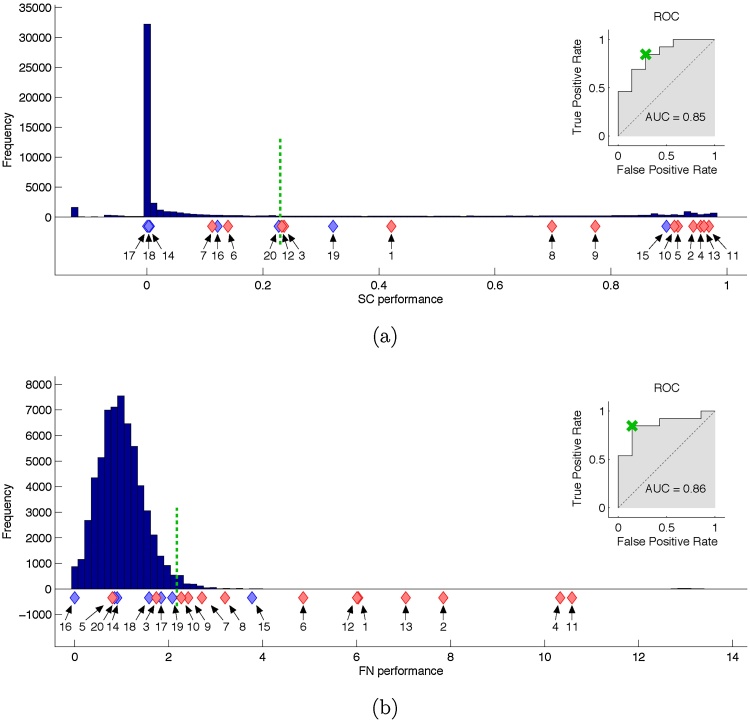
Assessments of random and actually carried out resections by the soft clustering (a) and the functional network (b) approach. Ratings of all patients’ random resections are accumulated in the histograms and ratings of actual carried out surgeries are shown beneath as red diamonds for class I patients or blue diamonds for class IV patients. The ROC-curves illustrate the methods’ performances as binary classifiers. The point on the ROC-curve with minimal distance to perfect performance (cross) determines the threshold of the optimal binary classifier (dotted vertical line). (For interpretation of the references to color in this figure legend, the reader is referred to the web version of this article.)

We first studied the performance of each method in terms of their ability to separate class I and class IV patients. We found that using the soft clustering approach the majority of the random resections did not considerably decrease the likelihood of the seizure states compared to no resection (most random resections are clustered towards 0 performance in Fig. 1a). The actual resections of all class I patients are unlikely to originate from this distribution (*p* = 5.9 * 10^−4^, test section B.3). In contrast, the actual resections of all class IV patients are very likely to originate from the distribution of random resections (*p* = 0.467, test section B.3). A notable outlier is patient 15 for whom the model wrongly predicts that the actual resection would be highly seizure prohibiting. We also found that some random resections had high performances. This is not surprising as it is very likely that resections other than the actual resection could have had a curative effect for the patient if performed. Class I and class IV patients are also significantly separated by the class-wise performances of the patients’ actual resections (*p* = 3.1 * 10^−4^, test section B.1). Using SC performance as a classifier, the area under the ROC curve is 0.85, indicating good patient-level classification.

Fig. 1b illustrates the ability of the functional network approach to separate class I from class IV patients. As for SC, most actual resections of class I patients were found to lie outside or at the very edge of the distribution of random resections (*p* = 5.7 * 10^−4^, test section B.3), whereas class IV patients showed strong overlap with this distribution (*p* = 0.294, test section B.3). Again, patient 15 was misclassified as having good response. For the FN measure, patient 5 was also clearly misclassified, as a poor, rather than a good, responder. Despite these two failures the method significantly separates class I and class IV patients by the class-wise performances of their actual resections (*p* = 1.6 * 10^−4^, test section B.1). The ROC analysis for the FN measure yielded an area under the ROC curve of 0.86.

**Fig. 2:**
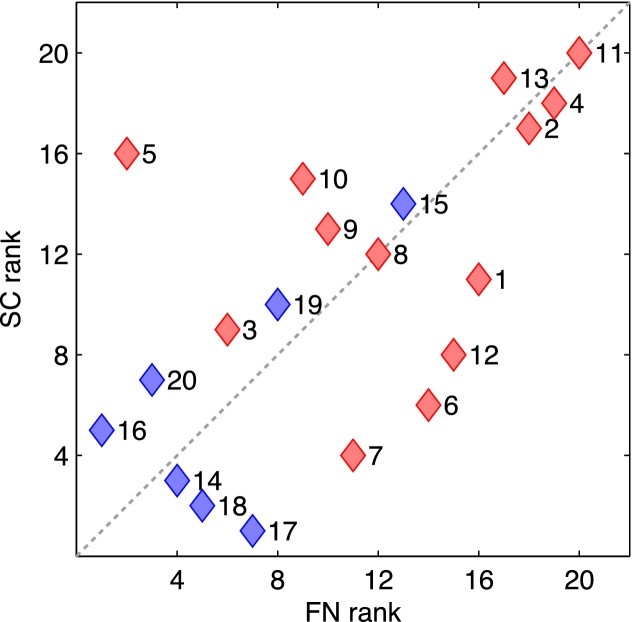
Comparison of the patients’ rankings by both methods. Red diamonds show class I patients while blue diamonds show class IV patients with the corresponding patient label to the right. The dotted diagonal indicates complete agreement between the ranking of both methods. The rankings of both methods correlate positively and significantly (Spearman's *ρ* = 0.60, *p* = 0.0027). (For interpretation of the references to color in this figure legend, the reader is referred to the web version of this article.)

In conclusion, both methods are individually able to distinguish class I from class IV patients by rating the actual resections and also by comparing them to random resections. In addition, the rankings of the patients by both methods correlate positively and significantly: Spearman's *ρ* = 0.60, *p* = 0.0027 (test section B.2). This correlation is visualized in Fig. 2.

We further determined the performances of the optimal binary classifiers of both methods and their combinations by AND- and OR-conjunction. The thresholds lie between patients 12 and 20 for the SC approach (see Fig. 1a) and between patients 10 and 19 for the FN approach (see Fig. 1b). The measures of all classifiers are given in Table 2. These thresholds additionally point out a considerable difference between the methods. While in the SC approach the random resections above this threshold account for about 25% of all random resections, it is only about 2% in the FN approach.

**Table 2:** Binary classifier performances.

	SC	FN	AND-conj.	OR-conj.
False negative	2	2	4	0
False positive	2	1	1	2

Sensitivity	0.85	0.85	0.69	1.0
Specificity	0.71	0.86	0.86	0.71
PPV	0.85	0.92	0.90	0.86
NPV	0.71	0.75	0.60	1.0

Classification errors and corresponding measures for the separate optimal binary classifiers of both methods and their combinations. Abbreviations: PPV: positive predictive value, NPV: negative predictive value.

**Fig. 3:**
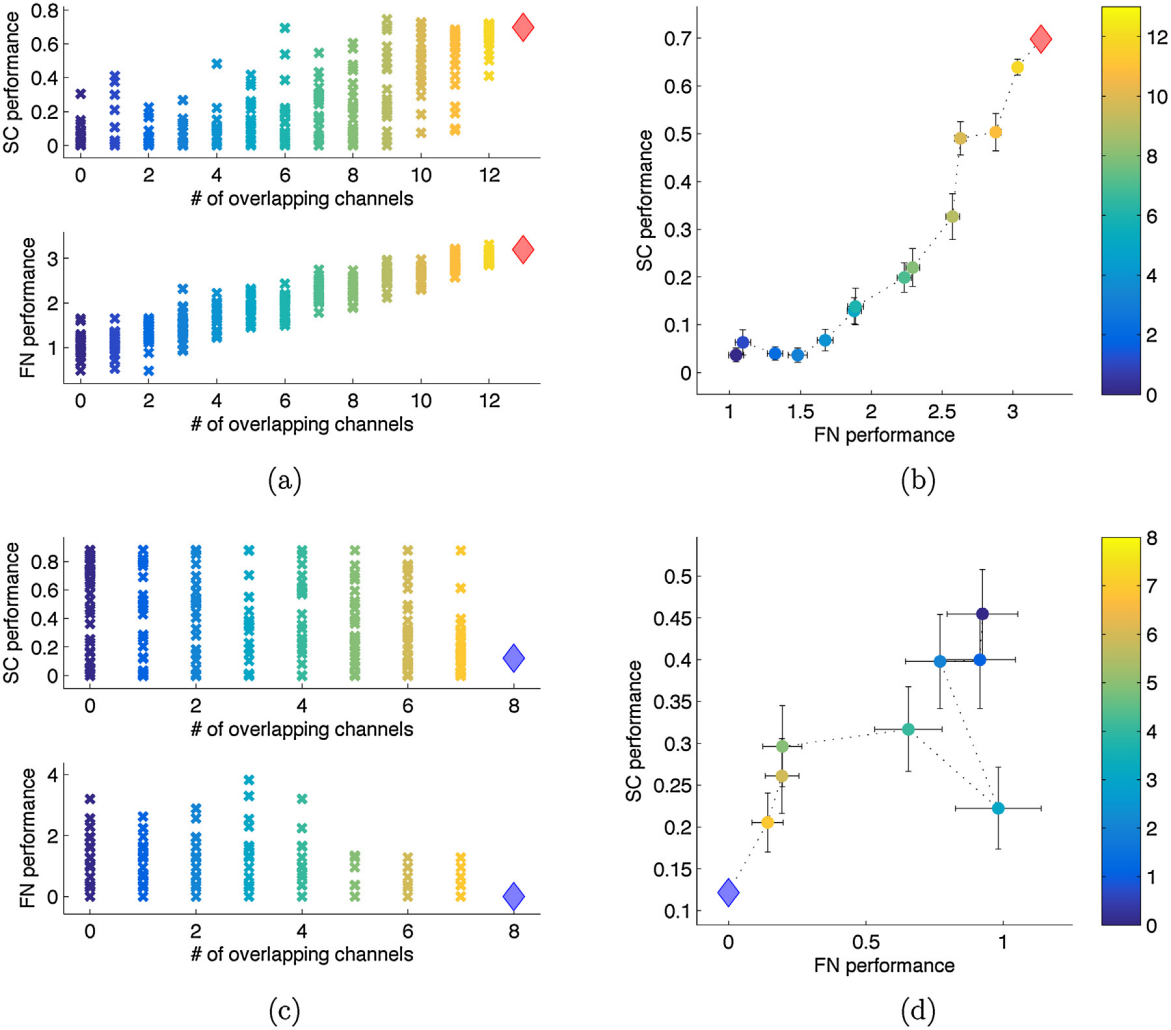
Single patients’ evaluation of random resections having a defined overlap with the actual resection. Panels (a) and (b) show the results for class I patient 8. Panel (a) shows the separate ratings of all 300 virtual resections by both methods (top: SC, bottom: FN). The overlap of the random resections with the actual resection is indicated on the *x*-axis and also color coded. The actual resection is shown as diamond. Panel (b) shows the group-wise means of both methods with errorbars indicating the standard error of the mean and the same color coding for the overlap as in panel (a). Panels (c) and (d) show the same for class IV patient 16. (For interpretation of the references to color in this figure legend, the reader is referred to the web version of this article.)

Next, we analyzed the methods’ dependences on a random resection's overlap with the actual resection. We grouped random resections according to the size of their overlap with the patient's actual resection and evaluated how the methods’ assessments are related to this overlap. Fig. 3 shows the results of one class I and one class IV patient. It is clear that both methods rate virtual resections with a larger overlap with a higher performance in the class I patient. However, no such dependence exists for the class IV patient. Panels (b) and (d) again indicate a relation between the two methods. Whereas the methods’ common positive trend (increasing performance with increasing overlap) observable in panel (b) appears in most class I patients, the negative trend (decreasing performance with increasing overlap) observable in panel (d) is not a general characteristic of class IV patients.

We quantified the relationship between overlap and performance using the correlation coefficient for each patient. Results are shown in Table 3. The null hypothesis that both classes have the same mean correlation coefficient, can be rejected for the soft clustering approach (*p* = 0.0403, test section B.4) and for the functional network approach (*p* = 0.0051, test section B.4). The high correlations of most class I patients in the functional network approach are induced by the fact that this approach's rating of a set is the fraction of node strengths comprised by the channels in this set. For this reason it is inherent to the functional network approach that ratings of sets change gradually with cumulative alterations. Thus, the more channels of the actual resection do have among the highest node strength values, the more likely any additive exchange of channels will cause the set's fraction of total node strength to decrease. This consequently induces a positive correlation between overlap and ratings. Hence, these results confirm that the functional network approach has a strong tendency to assign high node strengths to actually resected channels in class I patients.

**Table 3:** Pearson's correlation coefficient between the rating of random resections and their overlap with the corresponding actual resection.

Patient	Class	CC SC	CC FN
1	I	0.29	0.94
2	I	0.20	0.91
3	I	−0.07	0.33
4	I	0.59	0.76
5	I	0.60	−0.25
6	I	0.61	0.94
7	I	0.22	0.89
8	I	0.75	0.93
9	I	0.39	0.58
10	I	0.32	0.71
11	I	0.59	0.93
12	I	−0.02	0.97
13	I	−0.18	0.76
14	IV	−0.16	−0.02
15	IV	0.55	0.94
16	IV	−0.23	−0.40
17	IV	−0.41	0.46
18	IV	0.04	0.31
19	IV	0.59	0.64
20	IV	0.07	−0.14

Classes have significantly different means in both, the soft clustering approach (*p* = 0.0403) and the functional network approach (*p* = 0.0051).

**Fig. 4:**
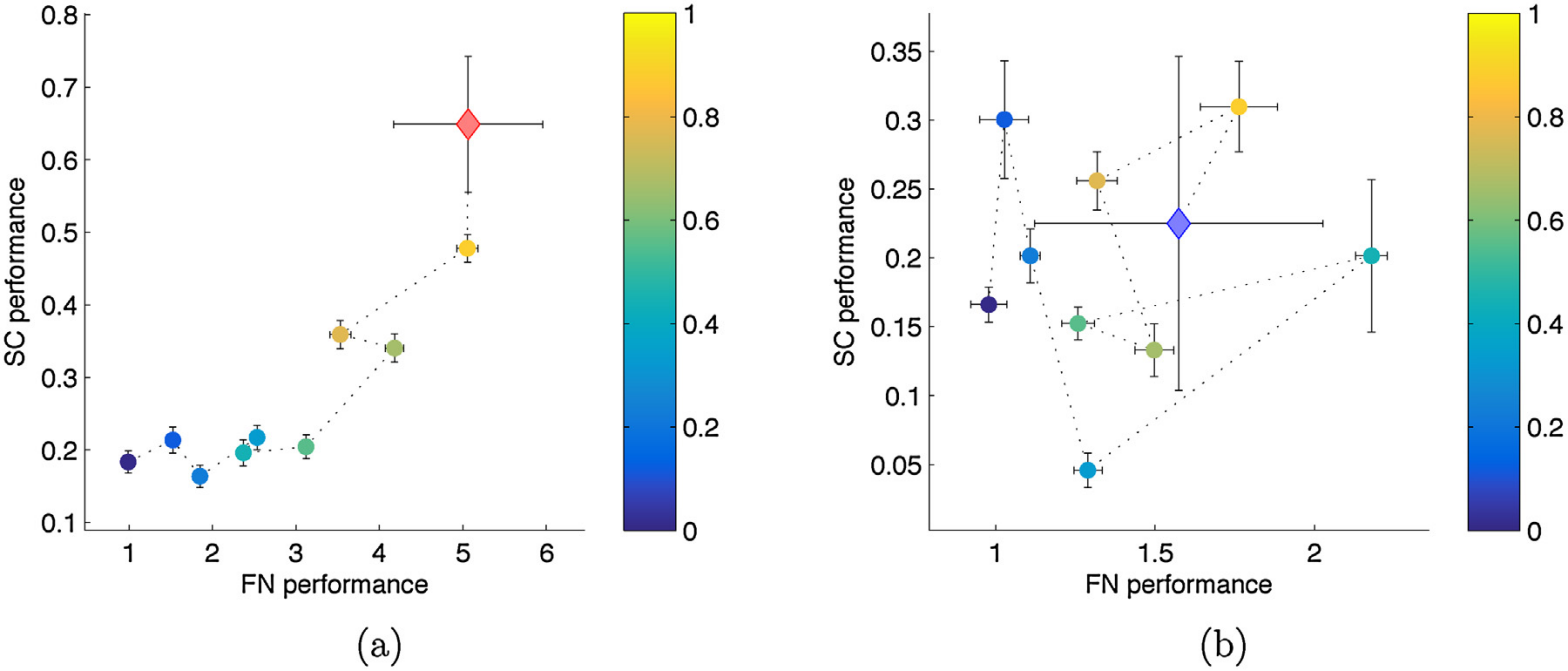
Comparison of both methods’ evaluations of all random and actual resections of all patients, assembled class-wise and grouped by their overlap with the corresponding actual resection. All random resections of all patients in an outcome class are split into nine bins according to their overlap as fraction of the respective patient's actual resection. The bin-wise means of both methods’ ratings are shown with the corresponding overlap color coded. The mean of the class’ actual resections is shown as diamond (red for class I and blue for class IV). Errorbars indicate the standard error of the mean. Panel (a) shows the relation of both methods in class I patients and panel (b) the same for class IV patients. (The larger errorbars for the groups of actual resections compared to those of random resections is due to the much smaller number of data points in the groups of actual resections.) (For interpretation of the references to color in this figure legend, the reader is referred to the web version of this article.)

Fig. 4 visualizes the results for class I and IV patients grouped separately. For class I patients, a significant correlation between the overlap of random resections (with the actual resection) and their rating exists in the functional network approach (*ρ* = 0.9707, *p* = 0, test section B.5) and in the soft clustering approach (*ρ* = 0.8584, *p* = 6.5 * 10^−4^, test section B.5). For class IV patients, the ratings of the soft clustering approach do not show significant correlation with the overlap of virtual resections (*ρ* = 0.2476, *p* = 0.245, test section B.5), while the ratings of the functional network approach do correlate significantly with the overlap of virtual resections (*ρ* = 0.5619, *p* = 0.046, test section B.5). This correlation is obviously induced by the strong correlations of patients 15 and 19 (Table 3) since without them the significant correlation disappears (*ρ* =−0.3199, *p* = 0.788, test section B.5). Accordingly, the ratings of the two methods significantly correlate positively for class I patients (*ρ* = 0.8748, *p* = 8.2 * 10^−4^, test section B.5), whereas for class IV patients the same cannot be stated (*ρ* = 0.1303, *p* = 0.366, test section B.5).

When the overlap is used as an explanatory variable for the ratings of each method and the correlation is calculated on the residuals, the significant correlation between the methods’ ratings disappears also in the outcome class I group (*ρ* = 0.3369, *p* = 0.169, test section B.5). This suggests the ratings of the methods to be conditionally independent given the overlap of a hypothetical resection.

**Fig. 5:**
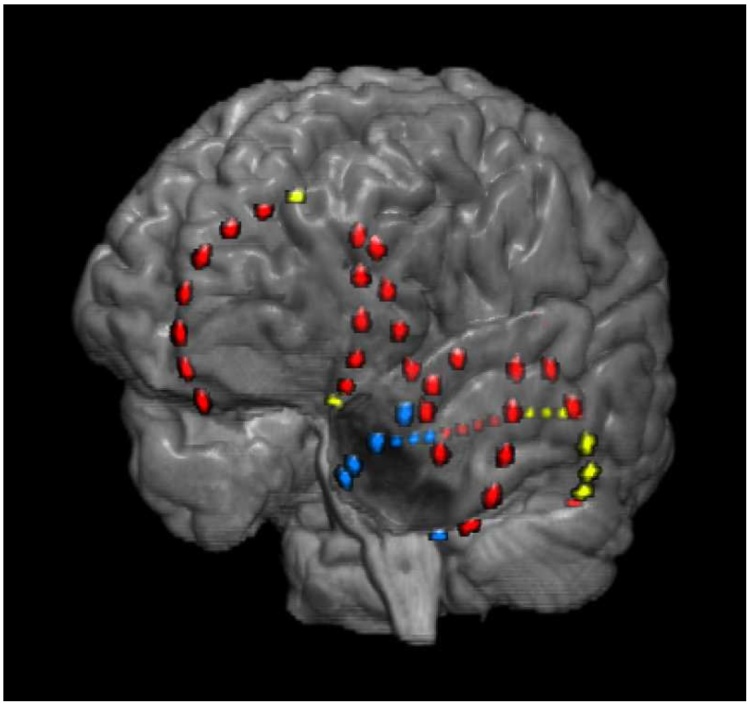
Representation of the actual resection of class IV patient 16 and a hypothetical resection assessed by both methods as highly beneficial. A pre-surgical MR recording was coregistered with a post-implantation CT recording do determine the position of the iEEG-electrodes (colored dots). The channels removed during the actual resection are located around the temporal pole (blue dots) whereas the hypothetical resection is mainly located in the posterior temporal lobe (yellow dots). (For interpretation of the references to colour in this figure legend, the reader is referred to the web version of this article.)

Although apparently the methods’ ratings do not generally coincide for resections not overlapping with a successful actual resection, such cases exist. In Fig. 5 we show a resection of class IV patient 16 that is assessed by both methods as highly beneficial and among the best random resections without overlap with the actual resection. Its performance values are 0.88 in the SC approach and 0.68 in the FN approach whereas this patient's actual resection has performance values of 0.12 (SC) and 0 (FN). While the actual resection was focused on the temporal pole, the methods’ selection targets mainly the posterior areas of the temporal lobe. This resection would however hardly be performed in reality because of possible compromise to the posterior language area (including Wernicke's area), something the quantitative methods do not account for at present. Irrespective of its overlap with eloquent cortex it is impossible to verify the benefit of such a hypothetical resection retrospectively, a fundamental limitation regarding the validation of quantitative methods we further discuss in Section 4.

## 4 Discussion

We analyzed the agreement of two methodologically entirely different methods’ assessments of possible epilepsy surgery targets. One method is based on functional network theory and estimates the nonlinear interrelation between EEG channels (here referred to as functional network approach). It defines EEG features that stand out from the background in a dynamic and data-driven manner. Thus, it assigns properties to time series but does not permit predictions for future time points where no information about the time series is available or if the underlying system is modified. The other method uses machine learning techniques to predict the likelihood of a seizure state (here referred to as soft clustering approach). A probabilistic clustering model for iEEG time series is derived which allows for predictions about the effects of resective surgery under controlled modulation. In particular, it provides the possibility to judge a set of virtually resected channels collectively instead of each channel separately. Despite their different procedures, both can be used to predict the effect of hypothetical resections.

The statistical claims in this study are limited by the restricted amount of data that was available and matched our inclusion criteria, especially the need to have knowledge about the post-surgical outcome for at least 1 year. To countervail this limitation we used bootstrapping in all hypothesis tests. Also, one should note that the fraction of resected electrode channels was significantly smaller in class IV patients compared to class I patients (test analog to Section B.1 using fractions of resected electrodes instead of ratings: *p* = 0.0197). This difference is consistent with the fact that in class I patients a better hypothesis of the SOZ could be generated based on non-invasive procedures before the implantation and thus the intracranial electrodes were more targeted towards this area. Having this in mind, it is not surprising that the fraction of resected channels also correlated with the methods’ ratings. While some data suggested a true relation between resection size and ratings (e.g. patient 15: large resection and high ratings despite class IV), others contradicted this assumption (e.g. patients 12 and 13: high ratings albeit small resections). Additional class I patients with small resections and/or class IV patients with large resections will be necessary to identify a potentially true, unbiased relation between resection size and rating. To test for a possible influence by the area of seizure spread, we determined the fraction of channels to where the ictal activity propagates during the seizure. If a channel showed epileptiform activity at least 10% of the total seizure time according to a procedure described in Schindler et al. (2007) it was considered as involved in the seizure. The fraction of involved channels cannot be separated significantly outcome-class-wise (test analog to Section B.1 using fractions of involved channels instead of ratings, *p* = 0.112), nor do they significantly correlate with the fractions of channels actually resected (test analog to Section B.2 using fractions of involved and resected channels instead of rankings, *p* = 0.302) or the assessments of either method (test analog to Section B.2 using fractions of involved channels and each method's rating instead of rankings, both *p* > 0.65). Thus, we conclude the seizure spread to have no relation with the outcome, the size of the actual resection or the ratings of the examined quantitative methods.

First, we compared the ability of each method to correctly assess actual resections. Both methods were able to separate class I and class IV patients by the ratings of the actual resections and their probabilities to originate from the distribution of random resections’ ratings (Fig. 1). In addition, the ranking of patients according to the performances of their actual resections correlated positively and significantly between the two methods (Fig. 2). We also defined the optimal binary classifier of both methods and compared their separate performances to their combined performances to determine a potential benefit from combining multiple quantitative methods. In general, the false positive (false negative) rate of an AND-conjuncted (OR-conjuncted) classifier is at most the lowest value of the separate classifiers, and thus its specificity (sensitivity) is at least as good as the best separate specificity (sensitivity). An AND-conjuncted classifier is thus rather preferable if the individual classifiers have high sensitivities but low specificities and vice versa, an OR-conjuncted classifier is rather preferable if the individual classifiers have high specificities but low sensitivities. In our case, no clear tendency to one or the other situation is observable. If a low false negative rate is important the OR-conjuncted classifier would be the obvious choice. Likewise, if a low false positive rate is important the best classifier would be the one by the FN method. One could also set the individual methods’ thresholds to yield perfect sensitivity or specificity and then combine them by the designated conjunction (see above). While this approach maximizes one of the two measures it completely disregards the other. Consequently, this procedure results in classifiers with unbalanced behavior and we did not notice distinct advantage from using it (results not provided). However, also due to the methods’ correlated rankings, the differences between all examined classifiers are small and preferences could easily change with additional patients. At this point, this Boolean combination of the methods does not have an evident beneficial effect on their decisive performance.

We further compared the ratings of arbitrary resections in terms of their overlap with the patient's actual resection. In general, for both methods, virtual resections with a larger overlap had better ratings if the actual resection rendered the patient seizure free (class I). If the actual resection had no beneficial effect for the patient (class IV), this relation became significantly weaker. Thus, in both methods the ratings of virtual resections were generally influenced by the overlap with the actual resection and its outcome (Figs. 3 and 4 and Table 3). In the soft clustering approach this dependence occurred particularly for large overlaps. Partial correlation analysis with overlap as controlling variable, however, suggested conditional independence of both methods.

Nevertheless, the methods also agreed on the misclassification of patient 15 who clearly showed the behavior of a class I patient in all tests (including the clinical assessment on which the surgery was planned), although in reality the surgical intervention did not have any beneficial effect. This suggests a connection between both quantitative methods that goes beyond the recognition of successful actual resections as effective and the dependence on the overlap with these resections. However, there were also some disagreements between the methods. Most prominently was patient 5 (class I) who was a clear true positive in the soft clustering approach but a similarly clear false negative in the functional network approach (Fig. 1). Consequently, patient 5 is also the one clear discrepancy in the ranking analysis (Fig. 2). Disagreement does not necessarily invalidate the methods as their predictions may also be based upon different signal features. Another noteworthy difference between the methods is the portion of random resections lying above the threshold of the optimal binary classifier (Fig. 1). While their fraction is relatively low in the FN approach (about 2%) it is substantial in the SC approach (about 25%). Higher fractions may indicate rather low specificity (see discussion below) which is consistent with our findings in Table 2 although no random resections were integrated in this analysis.

There are many studies with the goal to assess hypothetical resections or directly predict their outcome based on quantitative iEEG analysis (see e.g. Urrestarazu et al., 2007, Bartolomei et al., 2008, Worrell et al., 2008, Jacobs et al., 2009, Jacobs et al., 2010, Kim et al., 2010, Kim et al., 2014, Wu et al., 2010, Jung et al., 2011, Modur et al., 2011, Wilke et al., 2011, Gnatkovsky et al., 2011, Gnatkovsky et al., 2014, Park et al., 2012, van Mierlo et al., 2013, Boido et al., 2014, Sinha et al., 2014, Sinha et al., 2016, Geier et al., 2015, Hutchings et al., 2015, Rummel et al., 2015, Zubler et al., 2015, Goodfellow et al., 2016, Steimer et al., 2017). However, all methods so far share the shortcoming that they have only been tested in a single study and although they have shown the potential to yield clinically relevant information, they are not yet applied in clinical routine. To raise further trust in such techniques and their assessments, they should be tested on larger sets of patients and, as in the present study, on their consistency among each other. This study addresses the latter problem by directly comparing two fundamentally differing methods to assess hypothetical resections based on iEEG recordings, using one common set of patients. The examined two methods show a high level of agreement despite their fundamentally differing techniques. As a consequence of the extensive agreement, a potential benefit of combining them is not identifiable. In general, the larger the agreement of different methods, the smaller is the potential performance increase by combining them. On the other hand, larger discrepancy among methods raises suspicion about their assessments and is therefore not desirable. Our results showing high agreement are encouraging and request further such studies to establish quantitative methods in the clinical preoperative process of epilepsy surgery.

One of the biggest impediments regarding an objective evaluation and comparison of such methods is the lack of a ground truth. For methods with the purpose to quantify the effect of hypothetical resective surgeries, it is obviously very crucial to quantify their correctness. However, the lack of a ground truth in terms of complete knowledge about the outcome of every hypothetical resection poses an inevitable challenge in this regard. In fact, the actual outcome of all possible surgeries except the one realized is unknown. Thus, only one true positive or one true negative result is known for every patient. This hinders the calculation of common evaluation measures such as sensitivity and specificity. Sensitivity in this scenario means that resections leading to seizure freedom in the patient if actually carried out are also classified as seizure prohibiting by a decisive method. Sensitivity determination can thus only be based on class I patients, where one true positive outcome is known. A more precise classification based on real data is hardly possible because no other resection with proven curative effect can be known. Specificity means that resections that would not render the patient seizure free if actually carried out are also classified as such by a decisive method. This is also difficult to determine as the only resections proven to be unhelpful are those carried out in class IV patients. Apart from the possibility to use the actual resections of class IV patients, one can compare the assessment of a class I patient's actual resection to random resections. Although there are probably other resections than the actual one that would have also had a curative effect, it is plausible to suppose that most random resections would have had no beneficial effect in reality. Hence, a large number of random resections resulting in a similar assessment as a successful actual resection is a strong indication for low specificity. Similar considerations apply to related measures like positive and negative predictive value and on the whole, the calculation of accuracies on this very limited amount of real data remains rather unsatisfying and thus an open issue.

## 5 Conclusion

In this study, we investigated the relationship between two quantitative iEEG methods regarding their predictions for the effects of resective epilepsy surgery. Both methods are individually able to distinguish successful surgeries from unsuccessful and random ones and based on the predicted effectiveness of performed surgeries, patients are ranked in a correlated order between the two methods. Further, we showed that the ratings of both methods typically depend on the number of channels in a virtual resection that is also present in a successful actual resection. In general, the methods came to the same assessment for most patients, even for one of the few misclassifications. We conclude that there is a connection between the ratings of these conceptually completely different methods, however, it is obviously not straight forward as the partial correlation analysis revealed. Thus, further research is needed to unravel the nature of this connection. Nevertheless, both methods can already provide clinically relevant information and support physicians in the presurgical evaluation process by enabling them to test their planned resection on its predicted effectiveness.

Provided positive evaluation on larger and unselected datasets, such methods could objectify and simplify the cumbersome preoperative process by providing automatically generated data. Additionally, they have the potential to reveal signal features and dynamics that are undetectable by expert EEG reading. However, the missing ground truth and its simultaneous necessity to validate such approaches poses a fundamental conflict. One possibility to improve on this problem could be the congruence of multiple methods, which was investigated here for fundamentally differing techniques.

## Conflict of interest

None of the authors have potential conflicts of interest to be disclosed.

## Figures and Tables

**Fig. 6:**
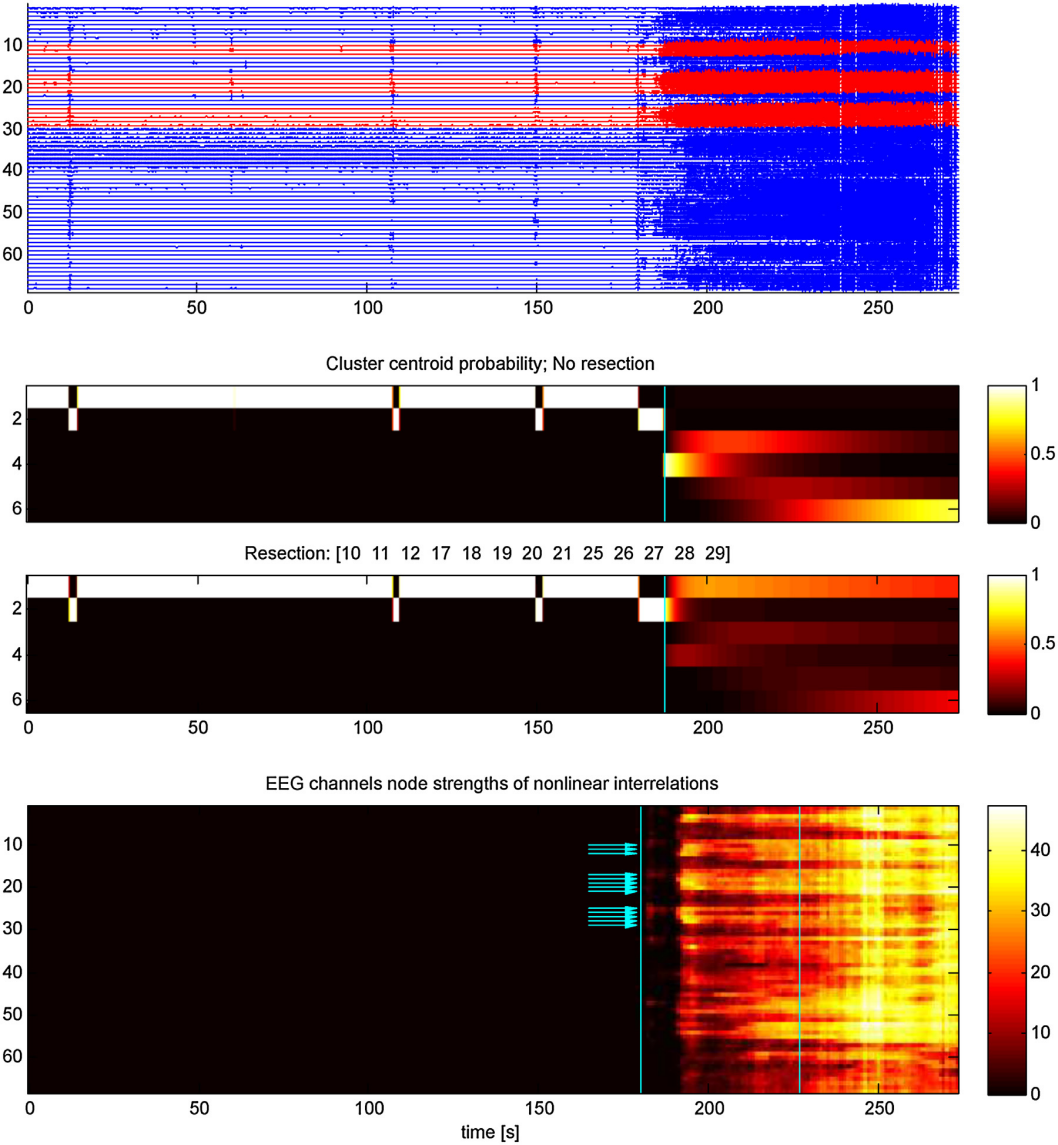
EEG data and evaluations of both examined methods of patient 8 (cf. Fig. 3a). Panel 1: Intracranial EEG recording. Clinically determined seizure onset is at 180 s and channels recording from tissue that was resected later are in red. Panels 2 and 3 show the evaluation of the soft clustering method. The probabilities of the *K* cluster centroids (*y*-axis) are shown over time, whereof clusters 3–6 are classified as ictal. The cyan lines indicate the time point where the input data was cut and subsequent centroid probabilities developed according to the model. Panel 2 shows the probabilities of the centroids if no resection is simulated and panel 3 shows the same if the actually performed resection is simulated. Panel 4 quantifies for each channel (*y*-axis, same order as in panel 1) the nonlinear interrelations over time. The cyan lines enclose the first half of the seizure, the segment used for evaluation, and the arrows indicate the actually resected channels. (For interpretation of the references to color in this figure legend, the reader is referred to the web version of this article.)

**Fig. 7:**
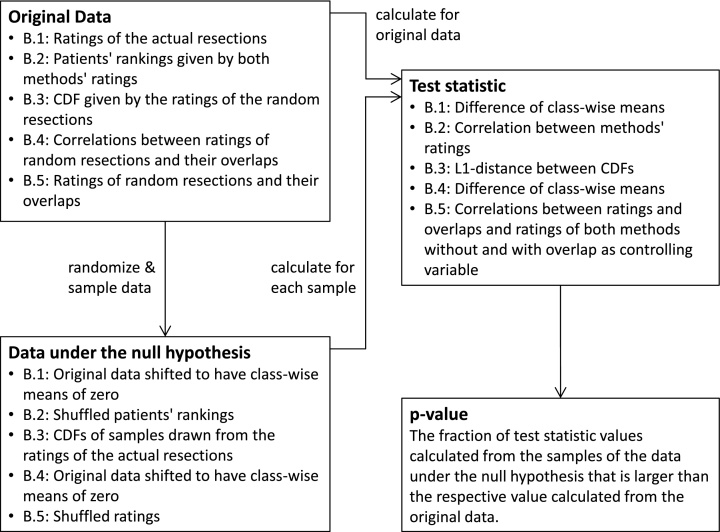
Flow chart summarizing all bootstrapping tests. The original data is modified in a specific way and 100,000 random samples are drawn with replacement to constitute the data under the null hypothesis. The same test statistic is calculated on the original data and on all randomized samples. The value of the original data in the distribution of the null hypothesis determines the *p*-value. Abbreviations: CDF: cumulative distribution function.

## Appendix A Quantitative methods in detail

This section describes for both examined quantitative methods the procedures carried out from an intracranial EEG recording to the final prediction about the efficacy of a particular resection of brain tissue associated with certain intracranial electrodes. The signal preprocessing was identical for both methods (see Section 2.1).

### A.1 Distributional soft clustering of multivariate time series

After bandpass filtering the EEG data, all channels were independently normalized to a mean of zero and a standard deviation of 1. The signals were then discretized to seven bins along the *y*-axis, with ±*σ* (SD) being the centers of the seventh and first bin (this corresponds to a bin width of *σ*/3). Values outside these bins were assigned to the nearest bin. Thus, a discretized recording of *n* channels has one out of *m* = 7^*n*^ states at every sampled instant. The discretized EEG time series were then partitioned by a moving window and all sampling points in a window, each being 1 of *m* states, were condensed into a single data point (feature). Such a feature is thus given by the *m*-dimensional empirical distribution of the states in that window. These distributions of all time windows were clustered into *K* = 6 clusters, being the regions in phase space (of possible distributions) where the system under study typically resides during different epochs of its temporal evolution. Each cluster centroid was represented by a graphical model, specifically by a Chow–Liu tree as second-order, distributional approximation (Chow and Liu, 1968). (This is why discretization of the signals was necessary, as Chow–Liu trees are defined for discrete data only.) Additionally, the temporal evolution of the probabilities of these *K* cluster centroids is specified by a Markov chain. Thus, the goal in the process of generating a model is to compute the cluster centroids, Markov chain parameters and the posterior probabilities of the cluster centroids. The generated model specifies for every time window in the recording the probability of every centroid to represent the current state. The summed probability of all centroids at any time point is always one and the Markov chain specifies expected future centroid probabilities through its set of transition probabilities. The model can now be used to predict probabilities of the centroids under various different conditions. On the one hand, the data of individual channels can be modified which directly alters the probabilities of the centroids to represent the data. On the other hand, the data of all channels can be cut at any time point inside the recording, leaving the future development of the centroid probabilities to the system. All specifications necessary to reproduce the model can be found in Steimer et al. (2017).

We used this approach to assess the effectiveness of simulated resections to prevent a developing seizure. Specifically, we used a peri-ictal recording to generate a model and classified the centroids as ictal or non-ictal according to their activities in the preictal and the ictal period of the recording. Moreover, we cut the data of all channels right at the beginning of the seizure (when ictal centroids are already highly probable), leaving the seizure to develop according to the model's predefined temporal evolution (Fig. 6, panel 2). Then, we set the data of the channels whose resection we wanted to simulate to zero (the middle bin) and compared the likelihood of the ictal centroids to the situation without simulated resection(Fig. 6, panel 3). A decrease in the summed probability of all ictal centroids corresponds to the model's prediction that this particular resection would decrease the patient's propensity to develop seizures when actually carried out.

### A.2 Multivariate nonlinear interrelation based functional networks

In this approach, the channels of the intracranial EEG recording constitute the nodes of a functional network. To define the network's edges, we used mutual information which is based on information theory and is not restricted to Gaussianity or linear dependence. Mutual information quantifies the deviation of the observed joint distribution of the amplitudes of two time series from the product of their marginal distribution (which would imply statistical independence). After bandpass filtering, the EEG data was partitioned by a moving window and the mutual information between all pairs of channels was calculated for every window giving the mutual information matrix ***μ***. To correct for the influence of linear correlation we used multivariate IAAFT (iterated amplitude adjusted Fourier transform) surrogates (Schreiber and Schmitz, 2000). These surrogate time series, generated for each window, have the same autocorrelations and the same cross-correlations as the original time series. However, any nonlinear structure is removed and their corresponding mutual information matrices ***μ***_***surr***_ can thus be used as a baseline compensating for the effects of linear signal interrelations. This baseline was subtracted from the original matrix ***μ*** to get the surrogate corrected mutual information matrix ***M*** (Eq. (3)). Here, 〈***μ***_***surr***_〉 is the median of the values obtained from the set of surrogate time series and *s* is a significance factor that is 1 if the original matrix element is significantly different from the corresponding elements of the surrogate matrices and 0 otherwise. Hence, ***M*** is a sparse matrix specifying only nonlinear interrelations between EEG channels:

(3) $$M=\frac{\mu -\langle \mu_{\mathrm{surr}}\rangle }{1-\langle \mu_{\mathrm{surr}}\rangle }*s$$

Since we wanted a single value per channel (to eventually assign one value to an arbitrary set of channels) we condensed the matrix ***M*** by calculating the average node strengths over time. If *n* is the number of channels and *T* is the number of time windows in the examined segment, ***M*** has the dimensions *n* × *n* × *T*. The node strength of a channel was derived by summing over the absolute values of all its interrelations (minus the interrelation with itself) (Fig. 6, panel 4), and to average over time we took each channel's mean over all time windows in the first half of the seizure (Eq. (4)). Channel *i* accordingly had the node strength *s*_*i*_:

(4) $$s_{i}=\frac{\sum_{t=1}^{T}\left(\sum_{j=1}^{n}(|M_{i,j,t}|)-1\right)}{T}$$

To use this approach to assess the effectiveness of simulated resections, we calculated the fraction of the total node strength comprised by a particular set of channels. This value is the relative predicted performance of this set to decrease the patient's propensity to develop seizures if the corresponding resection would actually be carried out.

## Appendix B Bootstrapping tests in detail

The essential idea of bootstrapping is to determine the significance of some statistical measure based completely on the empirical data. This has the advantage that no assumptions about the underlying distribution have to be made. It is also appropriate when the sample size is small and sporadic samples could distort its representation of the population. Both conditions, the unknown underlying distribution and the small sample sizes are present in our case. Hence, we chose to apply bootstrapping methods in all our significance tests. The basic concept of these methods is to generate the test statistics’ distribution exclusively from the distribution of the empirical data. To do so, we selected and, if required, modified the empirical data in a way that depends on the specific null hypothesis and is described below separately for every test. From each resulting data set, we independently drew *N*_*samp*_ = 100,000 random samples. In this context, *sample* means a set of values drawn with replacement from an original set and having the same size as the original set. Calculating the desired test statistic on these random and independent samples, gives an appropriate distribution of values under the null hypothesis. The same test statistic is also calculated on the original data. The fraction of random samples having a higher value than the actual data is the corresponding *p*-value.

This basic idea was applied in all performed significance tests. Subsequently, for every test a short description is given for how the data under the null hypothesis and the corresponding *p*-value were calculated. Additionally, Fig. 7 summarizes the procedure for all cases in a flow chart.

### B.1 Separation of the two outcome groups by their actual resections

The goal of this test was to examine for both methods separately if the ratings of the actual resections separate the groups of class I and class IV patients. We shifted the empirical distributions (the ratings of both outcome groups) so they both have a mean = 0 and drew randomly *N*_*samp*_ samples of each distribution. The differences in the means of the pairwise but independent samples constituted the distribution under the null hypothesis that both distributions have the same mean. The corresponding *p*-value was the fraction of random sample-pairs having a bigger difference in their means than the actual outcome groups.

### B.2 Correlation of patients’ rankings as given by their ratings

The goal of this test was to examine if the methods rank the patients in a correlated order according to the ratings of their actual resection. Hence, we independently drew *N*_*samp*_ samples of each empirical distribution (each method's ranking) to generate pairs of uncorrelated data which constituted the distribution under the null hypothesis that there is no correlation between the rankings of both methods. The corresponding *p*-value was the fraction of random sample-pairs having a bigger rank correlation coefficient than the actual rankings.

### B.3 Congruency of the distributions of the actual resections and the random resections

The goal of this test was to examine if the actual resections of an outcome class are likely to originate from the same distribution as the random resections. Hence, for both methods and both outcome groups separately, we generated *N*_*samp*_ independent pairs of samples of the empirical distribution (the ratings of actual resections) and measured the L1-based distances between the pairs’ cumulative distribution functions (CDF). These values constituted the distribution under the null hypothesis that two empirical distributions have the same source. In addition, we determined the average L1-based distance between these samples and the CDF given by the ratings of the random resections. The fraction of values in the distribution under the null hypothesis that was bigger than this average L1-based distance was the corresponding *p*-value.

### B.4 Class difference in correlation of random resection ratings and overlap

The goal of this test was to examine if, depending on the outcome of the patient, the ratings of random resections correlate differently with their overlaps (the number of channels that are also in this patient's actual resection). Hence, the means of both datasets (each containing the appropriate correlation coefficients of one outcome group) were set to zero and the differences between the means of *N*_*samp*_ independent pairs of randomly drawn samples constituted the data under the null hypothesis that both classes have the same mean correlation coefficient. Accordingly, the fraction of random sample-pairs having a bigger difference in their mean correlation coefficient than the actual datasets was the corresponding *p*-value.

### B.5 Group-wise correlation between overlap and ratings and partial correlation of ratings

The goal of these tests was to examine relations between ratings of resections and their overlaps with the actual resection. First, we determined for both methods and both outcome groups separately the relation between the ratings and the overlaps of all virtual resections. We randomly drew *N*_*samp*_ samples of each empirical distribution (the ratings and the overlaps) to generate pairs of uncorrelated data which constituted the distribution under the null hypothesis that there is no correlation between a methods’ ratings and the overlaps of random resections. Accordingly, the corresponding *p*-value is the fraction of random sample-pairs having a bigger correlation coefficient than the actual datasets. Then, we used the ratings of both methods as empirical distributions to examine in exactly the same way the correlation among the ratings of both methods. Finally we used the concept of partial correlation to excluded the possible influence of the overlap as a controlling variable by calculating the residuals of both methods’ ratings using the overlap as regressor. These residuals then were the empirical distributions to examine in the same way as before the correlation between the ratings but with the effect of the overlap removed.
